# Nusinersen Treatment of Children with Later-Onset Spinal Muscular Atrophy and Scoliosis Is Associated with Improvements or Stabilization of Motor Function †

**DOI:** 10.3390/jcm12154901

**Published:** 2023-07-26

**Authors:** Sally Dunaway Young, Jacqueline Montes, Allan M. Glanzman, Richard Gee, John W. Day, Richard S. Finkel, Basil T. Darras, Darryl C. De Vivo, Giulia Gambino, Richard Foster, Janice Wong, Steve Garafalo, Zdenek Berger

**Affiliations:** 1 Department of Neurology and Clinical Neuroscience, Stanford University School of Medicine, Palo Alto, CA 94305, USA; 2 Department of Rehabilitation and Regenerative Medicine, Columbia University Irving Medical Center, New York, NY 10032, USA; 3 Children’s Hospital of Philadelphia, Philadelphia, PA 19104, USA; 4 Center for Rehabilitation Services, Stanford Children’s Health, Stanford University, Palo Alto, CA 94305, USA; 5 Center for Experimental Neurotherapeutics, St. Jude Children’s Research Hospital, Memphis, TN 38105, USA; 6 Department of Neurology, Boston Children’s Hospital, Harvard Medical School, Boston, MA 02115, USA; 7 Departments of Neurology and Pediatrics, Columbia University Irving Medical Center, New York, NY 10032, USA; 8 Biogen, Maidenhead SL6 4AY, Berkshire, UK; 9 Biogen, Cambridge, MA 02142, USA

**Keywords:** children, motor function, nusinersen, scoliosis, spinal muscular atrophy

## Abstract

Nusinersen has been shown to improve or stabilize motor function in individuals with spinal muscular atrophy (SMA). We evaluated baseline scoliosis severity and motor function in nusinersen-treated non-ambulatory children with later-onset SMA. Post hoc analyses were conducted on 95 children initiating nusinersen treatment in the CHERISH study or SHINE long-term extension trial. Participants were categorized by baseline Cobb angle (first nusinersen dose): ≤10°, >10° to ≤20°, and >20° to <40° (no/mild/moderate scoliosis, respectively). Outcome measures included the Hammersmith Functional Motor Score—Expanded (HFMSE) and the Revised Upper Limb Module (RULM). Regression analysis determined the relationships between baseline scoliosis severity and later motor function. For children with no, mild, and moderate scoliosis, the mean increase in HFMSE from baseline to Day 930 was 6.0, 3.9, and 0.7 points, and in RULM was 6.1, 4.6, and 2.3 points. In the linear model, a 10° increase in baseline Cobb angle was significantly associated with a −1.4 (95% CI −2.6, −0.2) point decrease in HFMSE (*p* = 0.02) and a −1.2 (95% CI −2.1, −0.4) point decrease in RULM (*p* = 0.006) at Day 930. Treatment with nusinersen was associated with improvements/stabilization in motor function in all groups, with greater response in those with no/mild scoliosis at baseline.

## 1. Introduction

Spinal muscular atrophy (SMA) is a rare genetic progressive neuromuscular disease characterized by muscle weakness, fatigue, and atrophy [1]. SMA is caused by insufficient levels of survival motor neuron (SMN) protein, leading to loss of motor neurons in the anterior horn of the spinal cord [1,2]. SMA occurs in approximately one per 10,000 live births [3,4,5,6], and almost 40% of those with SMA have type II or III [1]. In the natural history of SMA without treatment, individuals with SMA type II typically develop symptoms between 6 and 18 months of age and are able to sit unsupported but are never able to walk independently [1]. Individuals with type III, a milder form of SMA, typically develop symptoms after 18 months of age and are able to walk independently but lose function and may eventually become non-ambulatory [1].

During the natural course of the disease, orthopedic problems are common among children with SMA [7,8], including scoliosis [9,10], defined as a curvature of the spine with a Cobb angle >10° [11]. In a natural history study, the mean age at the onset of scoliosis was approximately 4 years in individuals with intermediate SMA (type II) and approximately 10 years in individuals with mild SMA (type III) [12,13]. A linear correlation has been observed between scoliosis and age in individuals with intermediate SMA (type II) and those with mild SMA (type III) who lost the ability to walk [12].

Worsening scoliosis in individuals with SMA type II or IIIa can lead to a decline in pulmonary function and affect the ability to sit, stand, or walk, limiting the individual’s functional ability, and can also lead to the need for surgical intervention [14,15,16]. In a longitudinal study, 28 individuals with SMA type II who had scoliosis experienced decline in functional motor scores, as measured by the Hammersmith Functional Motor Scale—Expanded (HFMSE) at a rate of 2.15 points per year between the ages of 5 and 13 years [17]. A retrospective analysis of 84 patients with SMA type II found a positive correlation between age and scoliosis angle (*p* < 0.001), with a progressive increase in scoliosis with age and a progressive increase in scoliosis angle with decreasing HFMSE scores [13].

Treatment with nusinersen has been shown to improve or stabilize motor function across a broad spectrum of SMA populations, including infants, children, adolescents, and adults, in clinical trials and/or real-world settings [18,19,20,21,22,23,24,25,26,27,28]. In the phase 3, randomized, double-blind, sham-procedure–controlled CHERISH study in children with later-onset SMA (most likely to be classified as type II or III), treatment with nusinersen significantly improved motor function (compared with a sham procedure), with a favorable safety profile [25]. However, there are limited data in the literature on the relationship between scoliosis and subsequent changes in motor function in individuals with SMA who have received nusinersen or other disease-modifying therapies. The aim of this study was to evaluate the association between baseline scoliosis severity and motor function outcomes in non-ambulatory individuals with later-onset SMA treated with nusinersen. Post hoc analyses were conducted using data from CHERISH and the nusinersen long-term extension study, SHINE.

## 2. Methods

### 2.1. Standard Protocol Approvals, Registrations, and Patient Consents

The CHERISH (NCT02292537) and SHINE (NCT02594124) study protocols were approved by the independent review board or ethics committee at each site. CHERISH was and SHINE is being conducted according to the provisions of the Declaration of Helsinki and the International Conference on Harmonisation Good Clinical Practice guidelines. In accordance with institutional guidelines, written consent was obtained from the parents, legal guardians, or the participants, depending on their age. An independent data and safety monitoring board or the institutional review board/independent ethics committee provided oversight of the studies in collaboration with the sponsor.

### 2.2. Study Population and Outcome Measures

These analyses utilized data from children who were initially enrolled in the CHERISH [25] study and then transitioned to the SHINE study. The CHERISH study was a multicenter, randomized, double-blind, sham-controlled phase 3 trial of nusinersen. CHERISH enrolled 126 participants with SMA, aged 2–12 years at screening, with symptom onset after 6 months of age and who were non-ambulatory. Participants were randomly assigned to intrathecal administration of nusinersen at a dose of 12 mg (nusinersen group), or a sham procedure, on Days 1, 29, 85, and 274. The CHERISH study was terminated early because nusinersen demonstrated a statistically significant effect on the primary endpoint (mean change from baseline at Month 15 on the HFMSE) [29,30], compared with sham control in the prespecified interim analysis [25]. A full description of the methods of CHERISH has been published previously [25].

The SHINE study is an ongoing open-label extension study of nusinersen in participants who were enrolled in previous nusinersen trials. The objective of SHINE is to provide long-term data on the safety and efficacy of nusinersen. Children who transitioned from the sham control group in CHERISH received loading doses of nusinersen on Days 1, 29, and 85 in SHINE, while children from the active (nusinersen) group in CHERISH received doses on Days 1 and 85 and sham procedure on Day 29 (to maintain blinding during the completion of the CHERISH study). Thereafter, all children from CHERISH received maintenance nusinersen 12 mg every 6 months in SHINE and, subsequently, following a protocol amendment, received 12 mg every 4 months.

The study protocols required Cobb angle assessment by X-ray at screening in the CHERISH study and at screening and approximately yearly thereafter in the SHINE study (with the exception of sites in Germany, where X-rays to assess scoliosis were only performed if clinically indicated to comply with the standard of care). X-rays were performed on children while seated after the removal of braces and assistive devices. If required, assistance from a parent or technician in maintaining a seated position was permitted, without attempting to reduce the degree of scoliosis. Performing X-rays in a supine position was not permitted. Anatomical coverage included the thoracolumbar spine (C7–S1) with alignment of the mid-sagittal plane with the center of the film/cassette/receptor. Radiographs were centrally read (Parexel International). The normal variability in Cobb angle readings was expected to be 5–10° [31,32,33].

For these post hoc analyses, children who initiated treatment with nusinersen in SHINE (i.e., those from the CHERISH sham group) were re-evaluated using the eligibility criteria from the CHERISH study for inclusion in this analysis. These criteria included a total score of ≥10 to ≤54 on the HFMSE; age 2‒12 years; inability to walk independently (defined as the ability to walk ≥15 feet unaided), but ability to sit without support per World Health Organization (WHO) motor milestones [34]; and absence of respiratory insufficiency (i.e., use of invasive or non-invasive ventilation for >6 h/day), gastric tube for nutrition, and severe scoliosis, defined as curvature of the spine with a Cobb angle ≥40° at baseline. Additional criteria for all children evaluated (in this analysis of scoliosis impact on motor function) included completion of Cobb angle assessments at baseline and HFMSE assessment at baseline and Day 930 of nusinersen treatment.

Outcomes included motor function measures (HFMSE, Revised Upper Limb Module [RULM] [35], and WHO motor milestones) and the Cobb angle.

### 2.3. Statistical Analyses

SAS (SAS Institute, Cary, NC, USA) was used for the statistical analyses. For all analyses, participants were categorized by baseline Cobb angle into three subgroups: Cobb angle ≤10° (no scoliosis), Cobb angle >10° to ≤20° (mild scoliosis), and Cobb angle >20° to <40° (moderate scoliosis) [11,36]. An HFMSE response was defined as achievement of a minimal clinically important improvement of ≥3 points [37], and an RULM response was defined as a minimal clinically meaningful improvement of ≥2 points [38]. To provide a context for the trajectory of scoliosis compared with untreated individuals, change in Cobb angle for the CHERISH sham and active groups within the CHERISH study was described.

A windowing approach was utilized to account for the early termination of the CHERISH study, the gap between the end of the CHERISH study and enrollment in the SHINE study for some participants, and differing assessment (visit) schedules between the two studies. Study days were derived from the first day of dosing with nusinersen (in either CHERISH or SHINE). Post first dose, assessments were assigned to study visits as follows: study visits that took place >50 and ≤131 days from the first dose were labeled as a target value of Day 92, visits >131 and ≤211 days from the first dose were labeled Day 169, visits >211 and ≤302 days were labeled Day 253, those >302 and ≤400 days were labeled Day 350, those >400 days and ≤570 were labeled Day 450, and then study visits >X − 120 to ≤X + 120, where X begins at Day 690 and increases in 240-day increments. In a situation where greater than one assessment was assigned to a window for HFMSE and RULM, the mean was utilized as the value in presentations. For WHO and the Cobb angle, the closest assessment to the target value was utilized. The baseline was defined as the last non-missing assessment prior to the first dose of nusinersen. For patients treated with nusinersen in CHERISH, the baseline was defined using assessments from screening or before dosing at Day 1 in CHERISH, and, for patients treated with sham control in the CHERISH baseline, was defined using assessments during screening or prior to Day 1 dosing of nusinersen in SHINE.

The following covariates were considered for assessment based on their clinical relevance: baseline HFMSE; baseline RULM; age at first dose (years); *SMN2* copy number; age at symptom onset (months); sex (female vs. male); baseline weight; baseline ulnar length; and baseline Cobb angle. A stepwise regression procedure (forward selection method with α = 0.05 as the entry criterion) was used to identify variables with the greatest impact on change from baseline in HFMSE and RULM. The final selected model was tested for multicollinearity and included baseline HFMSE/RULM, age at first dose (years), age at symptom onset (months), and baseline Cobb angle. As some patients underwent scoliosis surgery during the analysis period, a sensitivity analysis was also conducted to exclude HFMSE/RULM results after surgery and impute any missing values using a multiple imputation method. The cutoff date for data in these analyses was 27 August 2019.

The Markov Chain Monte Carlo (MCMC) method was implemented under the multivariate normality assumption to impute the missing post baseline score. The imputation step here included the covariates: age at first dose, age at onset of symptoms, baseline Cobb angle and baseline HFMSE score, along with the corresponding post-baseline values for HFMSE. RULM was dealt with in a similar way in a separate step.

## 3. Results

### 3.1. Participants

Of the 126 children enrolled in the CHERISH study, 125 enrolled into the SHINE study. In total, 95 children commenced treatment with nusinersen in CHERISH or SHINE and were included in this post hoc analysis; they all met the demographic and clinical inclusion criteria and had a baseline Cobb angle assessment and an HFMSE assessment at baseline and Day 930 (Supplementary Materials, Figure S1). For data analysis, the 95 children were categorized into three subgroups according to Cobb angle/scoliosis at baseline (defined as the last assessment before the first dose of nusinersen). Of these 95 children, 25 had a baseline Cobb angle ≤10° (no scoliosis), 37 had a baseline Cobb angle >10° to ≤20° (mild scoliosis), and 33 had a baseline Cobb angle >20° to <40° (moderate scoliosis) (Table 1). Baseline characteristics were generally similar across the subgroups, although children with moderate scoliosis (Cobb angle >20° to <40°) were slightly older and had a higher weight at baseline than children with no scoliosis (Cobb angle ≤10°), while children with no scoliosis (Cobb angle ≤10°) had higher baseline HFMSE scores than those in the higher Cobb angle subgroups. A higher proportion of participants in the Cobb angle ≤10° subgroup had two *SMN2* copies than in the higher Cobb angle subgroups.

### 3.2. Motor Function

Motor function outcomes were analyzed according to the Cobb angle/scoliosis status of participants at baseline.

#### 3.2.1. HFMSE and RULM

**Figure 1:**
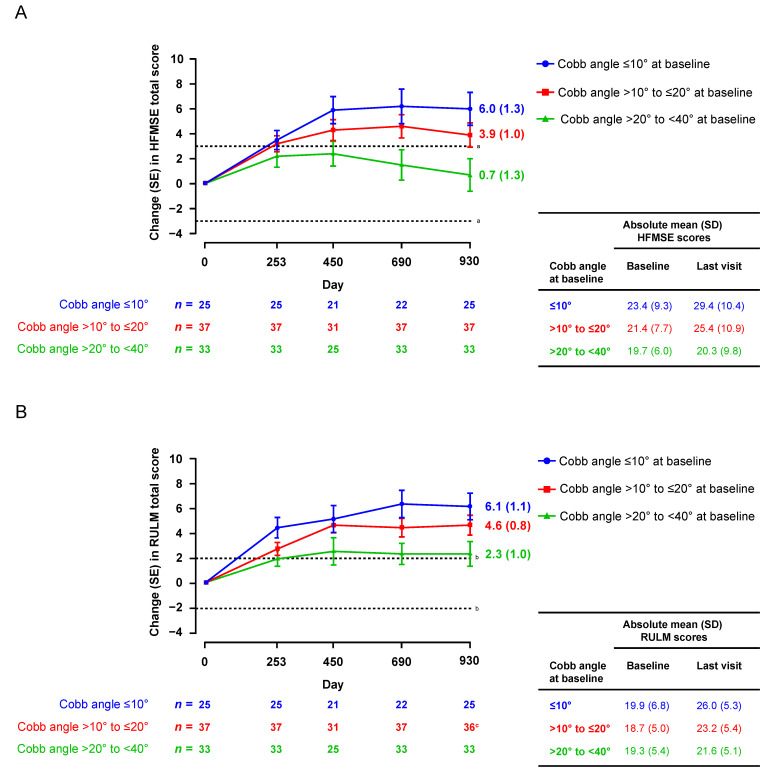
Motor function scores over time by Cobb angle subgroup: mean (SE) change in (**A**) HFMSE and (**B**) RULM. Data presented for visits with >10 participants. HFMSE, Hammersmith Functional Motor Scale—Expanded; RULM, Revised Upper Limb Module. ^a^ A ≥3-point change in HFMSE is considered clinically important [38]. ^b^ A ≥2-point change in RULM is considered clinically meaningful [39]. ^c^ *n* = 36 for RULM (one participant did not have an RULM assessment at Day 930).

**Figure 2:**
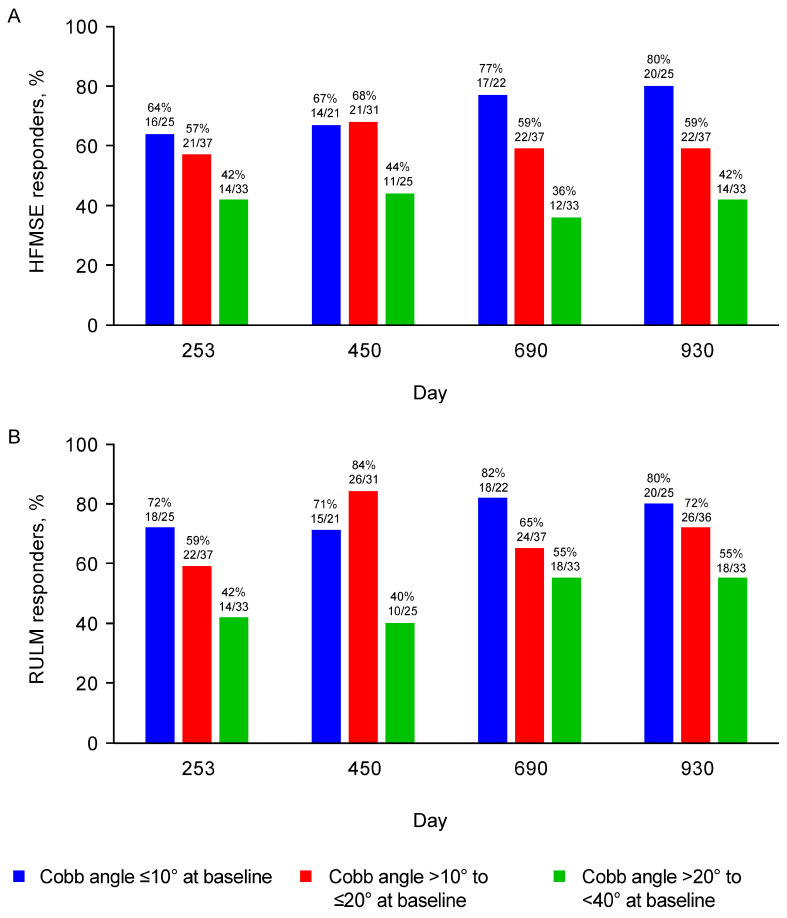
Proportions of (**A**) HFMSE and (**B**) RULM responders over time by Cobb angle subgroup. HFMSE response defined as improvement of ≥3 points; RULM response defined as improvement of ≥2 points. Data presented for visits with >10 participants. Not all participants had data that could be windowed to all visits shown; therefore, the denominators varied for some visits. HFMSE, Hammersmith Functional Motor Scale—Expanded; RULM, Revised Upper Limb Module.

Children without scoliosis at baseline (Cobb angle <10°) had the greatest mean improvement from baseline in HFMSE (6.0-point increase) and RULM (6.1-point increase) at Day 930 (Figure 1). In comparison to children without scoliosis, those with mild scoliosis (Cobb angle >10° to ≤20°) had a smaller mean improvement from baseline in HFMSE (3.9-point increase) and RULM (4.6-point increase) at Day 930. Children with moderate scoliosis (Cobb angle >20° to <40°) showed stabilization of HFMSE (0.7-point mean increase from baseline at Day 930) and RULM (2.3-point mean increase from baseline at Day 930). The greatest slope of improvement generally occurred between baseline and Day 450 across groups. Similar results were observed when assessing HFMSE and RULM responders over the same time frame (Figure 2). The proportions of HFMSE and RULM responders at Day 930 were 80% (20/25), 59% (22/37), and 42% (14/33), and 80% (20/25), 72% (26/36), and 55% (18/33) for participants with no, mild, and moderate scoliosis, respectively. In the sensitivity analysis that accounted for participants who underwent scoliosis surgery during the analysis period, similar results on HFMSE and RULM scores over time were observed.

#### 3.2.2. WHO Motor Milestones

**Figure 3:**
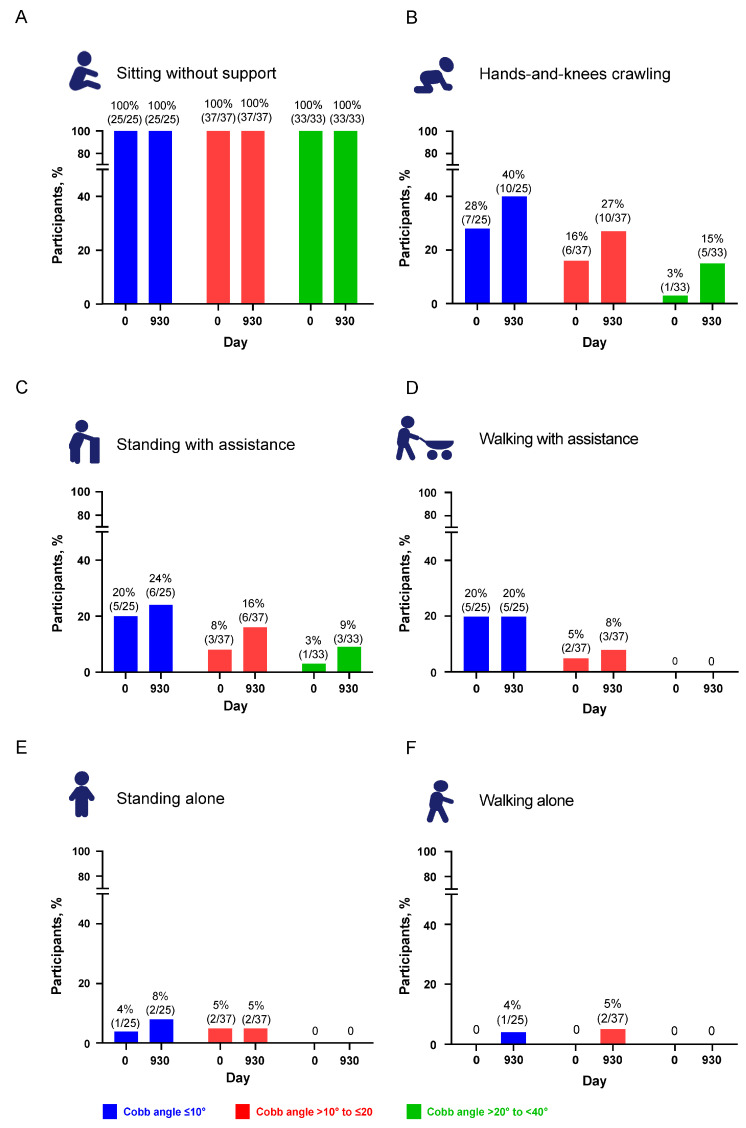
Proportions of participants achieving World Health Organization motor milestones at baseline compared with Day 930 by Cobb angle subgroup: (**A**) sitting without support, (**B**) hands-and-knees crawling, (**C**) standing with assistance, (**D**) walking with assistance, (**E**) standing alone, and (**F**) walking alone. Participants with Cobb angle ≥40° at baseline (last non-missing assessment prior to the first dose of nusinersen) were excluded.

The proportion of participants achieving WHO motor milestones at baseline and up to Day 930 was assessed according to scoliosis severity at baseline. In all subgroups and across all milestones, the proportion of participants achieving a given motor milestone was stable or higher at subsequent visits. Seven participants lost WHO motor milestones—three in the no scoliosis group, two in the mild scoliosis group, and two in the moderate scoliosis group. All except one child with no scoliosis at baseline had an increase in Cobb angle. Participants with no scoliosis (Cobb angle <10°) or mild scoliosis (Cobb angle >10° to ≤20°) were more likely to achieve WHO motor milestones of hands-and-knees crawling, standing with assistance, or walking with assistance by Day 930 than those with moderate scoliosis (Cobb angle >20° to <40°; Figure 3). At baseline and at Day 930, all participants were able to sit without support (Figure 3A). The proportion of children able to crawl on hands and knees increased across all three subgroups, with the proportion both at baseline and at Day 930 higher in subgroups with no scoliosis and mild scoliosis compared with the subgroup of moderate scoliosis (Figure 3B). The proportion of participants who were able to stand with assistance also increased across all subgroups, with the greatest increase among those in the mild scoliosis subgroup (Figure 3C). The proportion of participants achieving the WHO motor milestones of walking with assistance or standing or walking alone increased slightly or remained stable in the no scoliosis or mild scoliosis subgroups, whereas participants in the moderate scoliosis subgroup never achieved these motor milestones at any visit in the analysis (Figure 3E–F).

#### 3.2.3. Predictors of Motor Function Outcomes

A regression analysis was conducted to further assess the relationship between scoliosis severity at baseline and motor function. This analysis showed that baseline Cobb angle was significantly associated with later motor function outcomes (Table 2). The final selected model showed that an increase of 10° in baseline Cobb angle was significantly associated with a −1.4-point (95% CI −2.6, −0.2) decrease in HFMSE (*p* = 0.02) and a −1.2-point (95% CI −2.1, −0.4) decrease in RULM (*p* = 0.006) at Day 930. Multicollinearity was tested but was not observed in the final model. The sensitivity analysis, in which Day 930 results were estimated using a multiple imputation method to account for the exclusion of post-surgery results, showed that an increase of 10° in baseline Cobb angle was significantly associated with a −1.1-point (95% CI −2.2, 0.1) decrease in HFMSE (*p* = 0.08) and a −1.1-point (95% CI −2.0, −0.2) decrease in RULM (*p* = 0.01) at Day 930. In the sensitivity analysis, the upper CI estimate was slightly greater than zero for the HFMSE results, and for RULM, the estimates and CI were similar to the main analysis.

Age at first dose of nusinersen and age at SMA onset were also found to be significant predictors of later motor function for both HFMSE and RULM.

### 3.3. Changes in Scoliosis over Time

**Figure 4:**
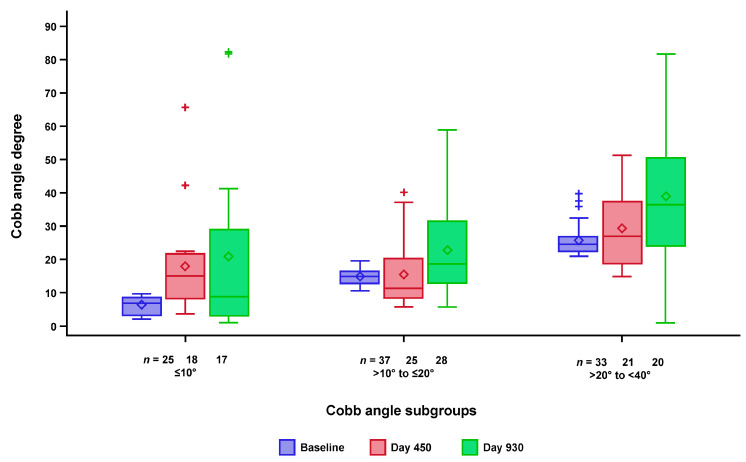
Boxplot of distribution of Cobb angle at baseline, Day 450, and Day 930 by Cobb angle subgroup. Outliers (+) are values outside 1.5 times the interquartile range above the 75th or below the 25th percentile range. Participants with Cobb angle ≥40° at baseline (last non-missing assessment prior to the first dose of nusinersen) were excluded.

To evaluate the longitudinal change in the degree of scoliosis over time (at baseline, Day 450, and Day 930), both the absolute values of the Cobb angles and change from baseline were analyzed descriptively. No apparent difference in scoliosis trajectories was seen among the three patient subgroups when analyzed descriptively. The distribution of Cobb angles at baseline, Day 450, and Day 930 for the three Cobb angle subgroups is shown in Figure 4. For all three subgroups, the variability in Cobb angle was greatest at Day 930. Over time, the mean Cobb angle increased in all three subgroups by age. It was noted that, at Days 450 and 930, the distribution was skewed, and the mean Cobb angle was greater than the median; this was particularly evident for the ≤10° subgroup, in which a few patients had increases in Cobb angle to >40° at later timepoints. Moreover, Cobb angle decreased for some participants, possibly attributable to variability in the assessment or following surgery.

Between baseline and Day 930, Cobb angle increased to ≥40° in 25 children, and 4 of the 25 underwent scoliosis surgery (spinal rod insertion, scoliosis surgery, or spinal fusion surgery). The majority were in the >20° to <40° (moderate scoliosis) Cobb angle at baseline subgroup. Of these participants, four developed a Cobb angle ≥40° by Day 450, and the remaining participants reached that level before Day 930. Characteristics of the four children and their HFMSE and RULM scores before and after surgery (as available) are provided in the Supplementary Materials, Table S1.

Analysis of Cobb angle data from the treated and sham control arms in the CHERISH study enabled a comparison between the treatment and natural history of SMA, albeit over a limited period of time. As a reference, the median change in Cobb angle from baseline of CHERISH to baseline of SHINE was 5.9° in the sham control arm and 1.2° in the nusinersen arm (Supplementary Materials, Figure S2).

## 4. Discussion

We evaluated the association between baseline scoliosis severity and motor function outcomes in non-ambulatory individuals with later-onset SMA treated with nusinersen by evaluating three subgroups: no scoliosis (a baseline Cobb angle ≤10°), mild scoliosis (baseline Cobb angle >10° to ≤20°), and moderate scoliosis (baseline Cobb angle >20° to <40°). All three subgroups exhibited improvements or stabilization in motor function with nusinersen treatment, regardless of scoliosis status at baseline. At baseline, children in the no scoliosis group had higher HFMSE scores and children in the moderate scoliosis group were slightly older and had a higher weight, but differences in baseline demographics and clinical characteristics were generally considered minor given their variability and were not associated with scoliosis severity.

Following nusinersen treatment, RULM scores increased in all three groups; HFMSE scores increased by ≥3 points in participants with no scoliosis and mild scoliosis and stabilized in those with moderate scoliosis. A higher degree of scoliosis in the moderate group may have limited performance on the HFMSE compared to the RULM, considering the broader range of motor functions evaluated with HFMSE. Participants with no scoliosis or mild scoliosis showed greater increase from baseline in HFMSE and RULM compared with those with moderate scoliosis. Participants with no scoliosis or mild scoliosis were more likely to achieve WHO motor milestones than those with moderate scoliosis. This is consistent with data showing that more advanced scoliosis in untreated patients is associated with impaired functional ability [12]. Similarly, baseline Cobb angle was a significant predictor of motor function outcomes at Day 930 (HFMSE, RULM). Overall, these data indicate that nusinersen treatment is associated with improvement and/or stabilization in clinical outcomes in patients across all three scoliosis groups, and baseline scoliosis severity is a predictor of motor function outcomes at later study visits.

Similar longitudinal trajectories in Cobb angle were seen over time for all three baseline Cobb angle subgroups. However, the data became increasingly variable over time, which was likely due to normal variations between readers and challenges in performing X-rays as participants grew with distribution skewed at Days 450 and 930. No formal indirect comparison to a natural history cohort was conducted to account for baseline differences, and the change in Cobb angle by CHERISH treatment groups occurred over too short a time to establish an annual rate, as seen in other studies. The annual rate of scoliosis progression (as observed via the Cobb angle) has been reported to range from 2.9–20° in SMA natural history studies [9,10,12,13]. In one study, the rate of scoliosis progression (change in Cobb angle) was 7.2° per year in 15 patients with SMA (type I, *n* = 1; type IIa, *n* = 12; type IIb, *n* = 2), with the most rapid progression (10.1°/year) occurring when scoliosis was more severe (18 months before spinal surgery) [9]. It should be noted, however, that patients in this natural history study had a median pre-operative Cobb angle of 50° [9], which represents more advanced scoliosis than the participants in this study. Such rapid progression prior to surgery is typical in patients with more advanced scoliosis (Cobb angle >40–50°). In another study exploring the natural history of scoliosis in SMA, the expected rate of progression (based on regression analysis) was 8.3° per year in 31 patients with intermediate SMA (type II) and 2.9° in 13 patients with mild SMA (type III but non-ambulant) [12]. In 18 patients with intermediate SMA (type II) who were followed for 4 months to 4 years, progression was 12° per year despite a brace, with the most rapid progression observed at approximately 10 years of age. Finally, another study reported an average annual progression of 20° in 18 patients with intermediate SMA (type II) who were non-walking (9° in four patients who were ambulant in orthoses), 5° in seven patients with mild SMA (type III) who were ambulant, and 15° in three patients with mild SMA (type III) who lost ambulation at puberty [10]. In this later study, scoliosis progression was rapid during puberty in those who lost ambulation [10].

Among individuals with intermediate SMA (type II) or non-ambulant mild SMA (type III), scoliosis progresses with age, although this association is not observed in those with mild SMA (type III) who are able to walk [12,40]. Worsening scoliosis affects limb movements, sitting, standing, gait, trunk stability, balance, bimanual activities, respiratory function, and activities of daily living; it also causes pain, impacting an individual’s functional abilities, independence, and quality of life [12,15,40]. The clinical course of SMA can now be altered with the availability of disease-modifying treatments [41]. The extent of the therapeutic response will likely depend on how soon treatment is initiated and the magnitude of pre-existing motor neuron degeneration [42].

Treatment in the pre-symptomatic stage, before considerable motor neuron loss, is ideal and underscores the importance of newborn screening protocols [43].

Early nusinersen treatment can impact a patient’s ability to gain new motor milestones and functional skills [20], as well as maintaining spinal flexibility and allowance for truncal compensatory mechanisms. Maintaining spinal flexibility can allow a patient’s center of gravity to be placed at the greatest mechanical advantage for weak muscles to function. Although scoliosis may not be completely preventable, for individuals with SMA who are receiving treatment, maintaining flexibility of the spine and extremities is functionally advantageous and can impact compensatory mechanisms in the presence of weakness.

These post hoc analyses of CHERISH/SHINE data have several limitations. The Cobb angle remains the gold standard for diagnosing and monitoring scoliosis [44]. However, it does not measure the amount of vertebral rotation or alignment of the spine or account for the degree of tilt on the endpoint vertebrae, and, therefore, is not the best indicator of the nature and severity seen in external appearance [33,44,45]. Additionally, the effect of pelvic obliquity on the Cobb angle was not taken into account in our study and, as the impact of contractures on function and scoliosis was not formally assessed until later in SHINE, the contribution of significant limb contractures to the decline in motor function was not measured or addressed as a possible confounding variable. Participants in this study had X-rays conducted in the seated position, as the majority of participants were unable to stand (with assistance or alone). Because assistance from a parent or technician was permitted, there could have been some variability in how the children were positioned for X-ray. Standard of care recommendations—including physical therapy, use of bracing (thoracic lumbar sacral orthosis), and standing devices—were not monitored. Most children in this analysis had not reached adolescence at the onset of scoliosis (mean age at first nusinersen dose was approximately 4 years) and were followed for up to 30 months, so those with severe scoliosis were not included in this analysis. Finally, as children with type II or III who were non-ambulant were included in these analyses, the impact of scoliosis on motor function in nusinersen-treated individuals with SMA type I, or those with SMA who are ambulant, has not been established.

Future studies should examine the trajectory of kyphosis in addition to scoliosis in patients with SMA, and the effect of its presence at baseline and on later clinical outcomes while on treatment. The impact of scoliosis on bilateral upper and gross motor function asymmetry should be explored among those who are treated. Furthermore, how the progression of scoliosis while on treatment affects significant comorbidities, such as contractures, as well as time spent sitting and standing, should be studied.

In summary, treatment with nusinersen was associated with improved or stabilized motor function over time across all three subgroups of participants with scoliosis from none to moderate. As expected, a trend toward greater clinical benefit was observed in children with lower (vs. higher) baseline Cobb angles. Our finding that baseline Cobb angle was a significant predictor of motor function outcomes in these post hoc analyses should be confirmed prospectively in future studies, as this may be useful in clinical practice.

## Figures and Tables

**Table 1:** Baseline characteristics.

Characteristic	Cobb Angle Subgroups			Total *N* = 95
	≤10° *n* = 25	>10° to ≤20° *n* = 37	>20° to <40° *n* = 33	
Female, *n* (%)	11 (44)	22 (59)	18 (55)	51 (54)
*SMN2* copy number, *n* (%) ^a^				
2	3 (12)	2 (5)	1 (3)	6 (6)
3	21 (84)	33 (89)	32 (97)	86 (91)
4	0 (0)	1 (3)	0 (0)	1 (1)
Median (range) age at first dose, years	4.0 (2.1–8.1)	4.0 (2.1–9.2)	4.4 (2.1–8.9)	4.2 (2.1–9.2)
Median (range) age at symptom onset, months	13.0 (7.0–18.0)	11.0 (6.0–18.0)	10.0 (6.0–20.0)	11.0 (6.0–20.0)
Cobb angle				
Mean (SD)	6.3 (2.64)	14.9 (2.69)	25.8 (4.83)	16.5 (8.46)
Median (range)	6.8 (2.1–9.8)	14.9 (10.4–19.6)	24.5 (20.9–39.8)	15.9 (2.1–39.8)
Mean (SD) weight, kg	14.8 (5.24)	15.3 (4.72)	15.6 (5.94)	15.3 (5.26)
Mean (SD) ulnar length, cm	14.8 (2.32) ^b^	14.8 (2.04) ^c^	17.5 (14.69) ^d^	15.8 (8.98) ^e^
Mean (SD) HFMSE score	23.4 (9.32)	21.4 (7.73)	19.7 (5.98)	21.4 (7.70)
Mean (SD) RULM score	19.9 (6.82)	18.7 (4.97)	19.3 (5.36)	19.2 (5.60)

HFMSE, Hammersmith Functional Motor Scale—Expanded; RULM, Revised Upper Limb Module; SD, standard deviation; *SMN2*, survival motor neuron 2. ^a^ Unknown for one participant with baseline Cobb angle ≤10° and one participant with baseline Cobb angle >10° to ≤20°. ^b^ *n* = 23. ^c^ *n* = 34. ^d^ *n* = 32. ^e^ *N* = 89.

**Table 2:** Regression model estimates and 95% confidence intervals of clinical motor function outcomes at Day 930 based on the final models selected.

Variables	Estimate (95% CI)	*p* Value
Change in HFMSE at:		
Intercept	12.86 (7.8,17.91)	<0.0001
Baseline HFMSE	0.04 (−0.09, 0.18)	0.52
Age at first dose of nusinersen	−2.69 (−3.27, −2.12)	<0.0001
Age at SMA onset	0.34 (0.04, 0.64)	0.03
Baseline Cobb angle	−0.14 (−0.26, −0.02)	0.02
Change in RULM at		
Intercept	16.2 (12.49, 19.91)	<0.0001
Baseline RULM	−0.32 (−0.46, −0.17)	<0.0001
Age at first dose of nusinersen	−1.63 (−2.11, −1.15)	<0.0001
Age at SMA onset	0.3 (0.08, 0.52)	0.008
Baseline Cobb angle	−0.12 (−0.21, −0.04)	0.006

CI, confidence interval; HFMSE, Hammersmith Functional Motor Scale—Expanded; RULM, Revised Upper Limb Module; SMA, spinal muscular atrophy. The following covariates (selected using a forward selection method) were used in the final model: baseline HFMSE/RULM, age at first dose (years), age at symptom onset (months), and baseline Cobb angle.

## Data Availability

Request for the data supporting this manuscript should be submitted to https://vivli.org/.

## Supplementary Materials

The following supporting information can be downloaded at: https://www.mdpi.com/article/10.3390/jcm12154901/s1, Figure S1: Participant flow; Figure S2: Boxplot of change from CHERISH baseline to SHINE baseline in Cobb angle by CHERISH treatment groups; Table S1: Characteristics of participants who underwent scoliosis surgery during the analysis period.

## Abbreviations

HFMSE: Hammersmith Functional Motor Score Expanded; RULM, Revised Upper Limb Module; SMA, spinal muscular atrophy; SMN, survival motor neuron; WHO, World Health Organization.
