# Supplementary material for: Nusinersen Treatment of Children with Later-Onset Spinal Muscular Atrophy and Scoliosis Is Associated with Improvements or Stabilization of Motor Function
Source: J Clin Med. 2023 Jul 26;12(15):4901. doi: 10.3390/jcm12154901 (PMC10419863; doi:10.3390/jcm12154901)
Supplement: Supplementary file 1 [file jcm-12-04901-s001.zip › jcm-2477026-supplementary.pdf]

# SUPPLEMENTARY MATERIALS

## Nusinersen Treatment of Children with Later-Onset Spinal Muscular Atrophy and Scoliosis Is Associated with Improvements or Stabilization of Motor Function

Sally Dunaway Young | Jacqueline Montes | Allan M. Glanzman | Richard Gee | John W. Day | Richard S. Finkel | Basil T. Darras | Darryl C. De Vivo | Giulia Gambino | Richard Foster | Janice Wong | Steve Garafalo | Zdenek Berger on behalf of the SHINE Study Group

**Supplementary Figure S1.** Participant flow.

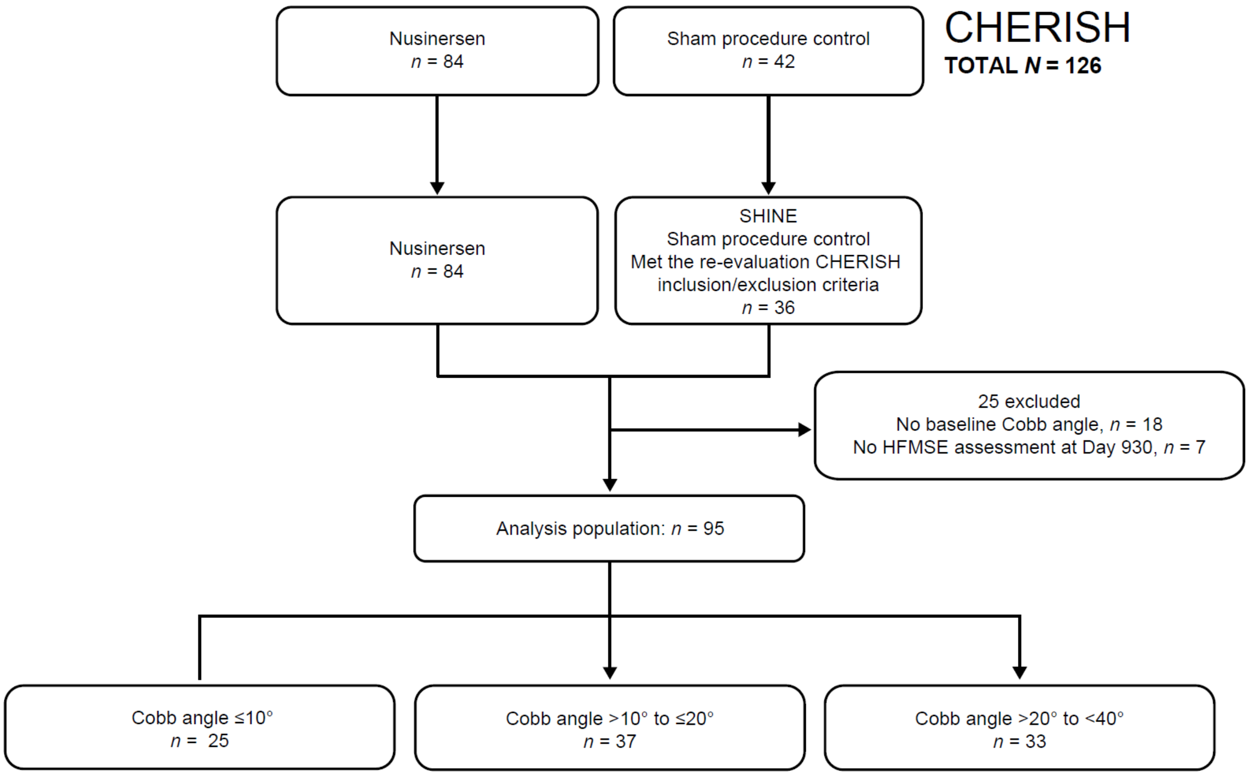


Participants previously on sham control were re-evaluated with CHERISH inclusion/exclusion criteria at SHINE baseline.

**Supplementary Figure S2.** Boxplot of change from CHERISH baseline to SHINE baseline in Cobb angle by CHERISH treatment groups.

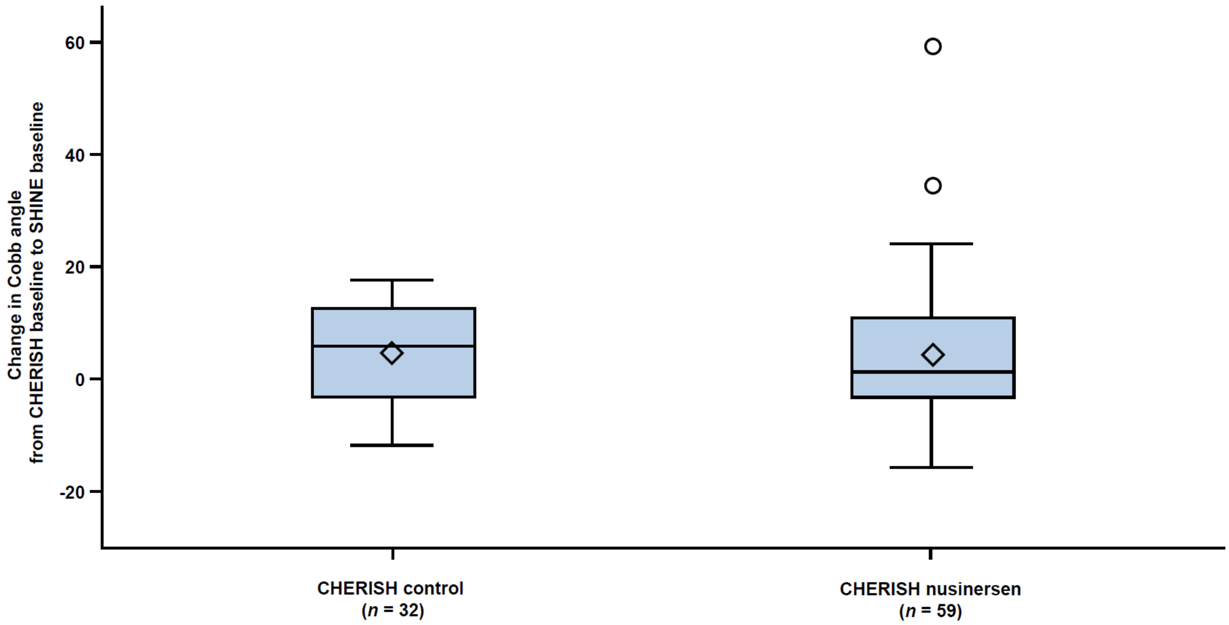


Outliers (o) are values outside 1.5 times the interquartile range above the 75th or below the 25th percentile range. Data are in participants with Cobb angle assessments at the baselines of both CHERISH and SHINE.

**Supplementary Table S1.** Characteristics of participants who underwent scoliosis surgery during the analysis period.

| | | Baseline[a] | | | Surgery | | HFMSE | | RULM | | Cobb angle | |
|---|---|---|---|---|---|---|---|---|---|---|---|---|
| Participant | *SMN2* copy number | Age, years | HFMSE | RULM | Day | Procedure/ reason | Before surgery (visit day) | After surgery (visit day) | Before surgery (visit day) | After surgery (visit day) | Before surgery (visit day) | After surgery (visit day) |
| 1 | 3 | 8.6 | 22 | 23 | 657 | Spinal fusion surgery/worsening of scoliosis | 20 (455) | 21 (734) | 21 (455) | 18 (734) | 44.5° (463) | 0.9 (916) |
| 2 | 3 | 6.4 | 13 | 16 | 715 | Scoliosis surgery/ progression of scoliosis | 13 (449) | 8 (750) | 15 (449) | 16 (750) | 22.8° (−18[b]) | 44.4° (926) |
| 3 | 3 | 5.2 | 17 | 23 | 871 | Spinal rod insertion/scoliosis | 15.5 (690) | 6 (930) | 19 (690) | 17 (930) | 67.8° (736) | 58.1° (1094) |
| 4 | 3 | 5.5 | 21 | 22 | 835 | Spinal Rod Insertion/Worsening of scoliosis | 14 (687) | 10 (930) | 22 (687) | 19 (930) | 69.5° (813) | NA |

HFMSE, Hammersmith Functional Motor Scale – Expanded; NA, not available; RULM, Revised Upper Limb Module; *SMN2*, survival motor neuron 2.

[a]Last non-missing assessment prior to the first dose of nusinersen.

[b]Before first nusinersen dose.

[c]Windowed to Day 930
